# Upregulated Expression of Cancer-Derived Immunoglobulin G Is Associated With Progression in Glioma

**DOI:** 10.3389/fonc.2021.758856

**Published:** 2021-10-25

**Authors:** Guohui Wang, Haonan Li, Jie Pan, Tianfang Yan, Huandi Zhou, Xuetao Han, Linlin Su, Liubing Hou, Xiaoying Xue

**Affiliations:** ^1^Department of Radiotherapy, The Second Hospital of Hebei Medical University, Shijiazhuang, China; ^2^Department of Central Laboratory, The Second Hospital of Hebei Medical University, Shijiazhuang, China; ^3^Department of Pathology, Stanford University School of Medicine, Stanford, CA, United States; ^4^Department of Neurological Diagnosis and Restoration, Osaka University Graduate School of Medicine, Suita, Japan

**Keywords:** cancer-derived immunoglobulin G, progression, glioma, microenvironment, IGHG1

## Abstract

**Objective:**

Gliomas are the most aggressive intracranial tumors accounting for the vast majority of brain tumors with very poor prognosis and overall survival (OS). Cancer-derived immunoglobulin G (cancer-IgG) has been found to be widely expressed in several malignancies such as breast cancer, colorectal cancer, and lung cancer. Cancer-IgG could promote tumorigenesis and progression. However, its role in glioma has not been revealed yet.

**Methods:**

We mined open databases including the Chinese Glioma Genome Atlas (CGGA), The Cancer Genome Atlas (TCGA), and the Gene Expression Omnibus (GEO) to study the role of *IGHG1*, which encodes cancer-IgG in glioma. Examination of the differential expression of *IGHG1* was carried out in the GEO and TCGA databases. Furthermore, its expression in different molecular subtypes was analyzed. Stratified analysis was performed with clinical features. Subsequently, immune infiltration analysis was conducted using single-sample gene set enrichment analysis (ssGSEA). GSEA was performed to reveal the mechanisms of *IGHG1*. Lastly, immunohistochemistry was processed to validate our findings.

**Results:**

In this study, we found that the expression of *IGHG1* was higher in glioma and molecular subtypes with poor prognosis. The overall survival of patients with a high expression of *IGHG1* was worse in the stratified analysis. Immune infiltration analysis indicated that the expression level of *IGHG1* was positively correlated with the stromal score, ESTIMATE score, and immune score and negatively correlated with tumor purity. Results from the GSEA and DAVID demonstrated that *IGHG1* may function in phagosome, antigen processing and presentation, extracellular matrix structural constituent, antigen binding, and collagen-containing extracellular matrix. Finally, immunohistochemistry assay validated our findings that patients with a high expression of cancer-IgG had poor OS and disease-free survival (DFS).

**Conclusion:**

Cancer-IgG is a promising biomarker of diagnosis and treatment for patients with glioma.

## Introduction

Gliomas are the most aggressive intracranial tumors accounting for the vast majority of brain tumors with very poor prognosis and overall survival (OS) (1). According to the malignant degree of glioma, the World Health Organization (WHO) classifies it into grades I–IV. Generally, grade I and II gliomas are considered less malignant and invasive. However, grades III to IV have a higher degree of malignancy and a strong invasive ability. In recent years, the diagnosis and evaluation of glioma have changed greatly, such as the combination of histopathological diagnosis and molecular markers. In 2021, the fifth edition of the WHO Classification of Tumors of the Central Nervous System (CNS) has been published. The latest classification emphasizes the importance of molecular and integrated diagnosis in the diagnosis and treatment of glioma (2). Standard therapy includes maximal safe tumor resection and radiation therapy with oral chemotherapy (3). Recent evidence found that tumor treating fields have good prospects for the treatment of gliomas (4). But the OS of patients with glioma is still very poor. Glioblastoma (GBM) patients have the worst prognosis, with a 5-year survival rate of less than 5% and, eventually, relapse (5). Therefore, it is urgent to explore new biomarkers for the treatment of glioma.

The classical immunological theory holds that immunoglobulin G (IgG), which plays a great role in human defense against pathogenic microorganisms, is produced only by B lymphocytes and plasma cells. However, more and more studies have shown that cancer cells can also produce IgG, called cancer-derived immunoglobulin G (cancer-IgG), such as those in breast cancer, colon cancer, cervical cancer, and lung cancer (6–9). Lee et al. immunized mice with the cleavage product of the ovarian cancer cell line OC-3-VGH to obtain the monoclonal antibody (mAb) RP215, which can specifically recognize cancer-IgG by recognizing a special glycosylation site in the constant region of the IgG heavy chain (10). Liao et al. found that cancer-IgG recognized by RP215 promoted the proliferation, invasion, and metastasis of tumor cells, which is a potential tumor stem cell marker (6). Tang et al. also showed that cancer-IgG promoted the occurrence and development of lung squamous cell carcinoma by activating the focal adhesion pathway (8). Some studies have also shown that cancer-IgG induced tumor immune escape by inhibiting effector T-cell proliferation in the tumor microenvironment (TME) (11). IgG consists of two heavy chains and two light chains. Each heavy chain and light chain is composed of a constant region and a variable region. The expression of IGHG1, which encodes the constant region of immunoglobulin heavy chain, is positively correlated with cancer-IgG (12). Accumulating evidence proved that IGHG1 is highly expressed in tumors and promotes oncogenesis and progression (13, 14). However, cancer-IgG and IGHG1 have not been studied in gliomas.

In this study, we firstly analyzed the expression of *IGHG1* in glioma and its relationship with prognosis through The Cancer Genome Atlas (TCGA), Gene Expression Omnibus (GEO), and the Chinese Glioma Genome Atlas (CGGA) databases. In addition, we also analyzed its possible mechanism through immune infiltration and gene set enrichment analysis (GSEA). Finally, the expression of cancer-IgG in glioma and its relationship with OS and progression-free survival (PFS) were analyzed by immunohistochemistry.

## Materials and Methods

### Data Acquisition and Processing

RNA sequencing and clinical data of patients with lower grade glioma (LGG) and GBMs were downloaded from TCGA database. We also obtained the gene expression profiling and corresponding clinical features of gliomas from the CGGA (15). The microarray dataset GSE4290 was downloaded from the GEO database (16). All RNA sequencing data downloaded from TCGA and CGGA should be standardized and batched by the R limma package. Excluding patients with unknown or incomplete clinicopathological parameters, only the gliomas with complete clinicopathological parameters and survival data in the dataset were retained.

### *IGHG1* Differential Expression Analysis

In the GSE4290 cohort, differences in the expression of *IGHG1* in glioma and normal brain tissues were analyzed. The expression levels of *IGHG1* in LGG, GBM, and normal brain tissues were compared in TCGA and GETx databases. Then, the patients were grouped according to the following clinical characteristics: age (≤41 and >41 years), gender (female or male), grade (grades II, III, and IV), status (alive or dead), isocitrate dehydrogenase (IDH) status (mutation or wild type), 1p19q status [co-deletion (codel) or non-codel], and *O*^6^-methylguanine-DNA methyltransferase (MGMT) (methylated or unmethylated). Wilcoxon tests were adopted to analyze the differential expressions between the abovementioned groups.

### Prognostic Analysis

Patients with glioma were divided into a high group and a low group according to the median value of the expression of *IGHG1*. Then, the patients were stratified according to their clinicopathological features, such as age (<42 and ≥42 years), gender (female or male), grade (grades II, III, and IV), IDH status (mutation or wild type), 1p19q status (codel or non-codel), and MGMT (methylated or unmethylated). Kaplan–Meier survival analysis was implemented to calculate the survival rates in the groups with low and high expressions of *IGHG1*. Then, we analyzed the relationship between the expression of *IGHG1* and progress-free interval (PFI) in TCGA cohort.

### Evaluation of the Effect of *IGHG1* on Glioma Microenvironment

Single-sample gene set enrichment Analysis (ssGSEA) was used to estimate the population fractions of immunocytes in gliomas. In addition, the degree of immune cell infiltration was quantified using enrichment scores calculated through the Gene Set Variation Analysis package of the R software. According to the degree of immune cell infiltration in the TME, glioma patients were divided into high, medium, and low immune groups. Spearman’s correlation analysis was used to analyze the relationship between the expression of *IGHG1* and the tumor purity, stromal score, ESTIMATE score, and immune score.

### Database for Annotation, Visualization and Integrated Discovery and GSEA

The differentially expressed genes (DEGs) between gliomas with high and low expressions of *IGHG1* in the CGGA cohort were identified using the R limma package and the following criteria: |logFC| > 1 and false discovery rate (FDR) <0.05. In order to further explore the possible mechanism of *IGHG1*, we conducted Gene Ontology (GO) and Kyoto Encyclopedia of Genes and Genomes (KEGG) pathway analysis using the Database for Annotation, Visualization and Integrated Discovery (DAVID) 6.8 online website (https://david.abcc.ncifcrf.gov/). The GSEA 4.0.2 software was also used for this purpose. A normalized enrichment score (NES) >1 and FDR <0.05 were considered meaningful.

### Immunohistochemical Staining and Scoring

The tissue microarray (TMA) used in this study was purchased from Shanghai Outdo Biotech Co., Ltd. (Shanghai, China). All patients were diagnosed with glioma by pathology. The mAb RP215 (sc-69849; Santa Cruz Biotechnology, Santa Cruz, CA, USA) was used to specifically recognize cancer-IgG. Human tissues were stained using mouse and rabbit specific horseradish peroxidase (HRP)/3,3′-diaminobenzidine (DAB) Detection IHC Kit (ab64264; Abcam, Cambridge, UK) according to the manufacturer’s instructions. The immunohistochemical staining score was based on previously published articles. The staining intensity was scored as follows: 0: no staining; 1: weak staining; 2: moderate staining: and 3: strong staining. The positive staining cell rate was scored as follows: 0: 0%–5%; 1: 5%–25%; 2: 26%–50%; 3: 51%–75%; and 4: >75%. A score below three points was considered negative and more than three points as positive.

### Statistical Analyses

The levels of *IGHG1* in tumor and normal tissue samples were compared using the Wilcoxon signed-rank test. Spearman’s rank correlation coefficients between *IGHG1* expression and the tumor purity, stromal score, ESTIMATE score, and immune score were tested. Kaplan–Meier survival curves were used to analyze the effect of *IGHG1* and tumor-derived IgG on prognosis. Statistical analyses were performed using IBM SPSS 24.0, GraphPad Prism 6, and R 4.0.1 software. *P* < 0.05 was considered statistically significant.

## Results

We utilized open databases to explore the expression of *IGHG1*, which encodes the heavy chain of IgG. There are 176 cases in the GSE4290 dataset, 587 in TCGA, and 686 in the CGGA database. Besides, a TMA containing 169 cases was included in our study. The clinical characteristics and molecular features are all listed in **Table 1**.

**Table 1:** Clinicopathological characteristics of glioma patients from the Gene Expression Omnibus (GEO), The Cancer Genome Atlas (TCGA), and the Chinese Glioma Genome Atlas (CGGA) databases and tissue microarray.

	GSE4290 (*n* = 176)	TCGA (*n* = 587)	CGGA (*n* = 686)	Tissue microarray (*n* = 169)
Age (years)				
<42	NA	242	308	37
≥42	NA	345	378	132
Gender				
Female	NA	246	287	62
Male	NA	341	399	107
Normal tissue	23	NA	NA	NA
Grade				
II	45	211	177	97
III	31	234	226	51
IV	77	142	283	21
IDH status				
Wild type	NA	219	315	NA
Mutation	NA	368	371	NA
1p/19q				
Codel	NA	149	141	NA
Non-codel	NA	438	545	NA
MGMT				
Methylated	NA	NA	386	NA
Unmethylated	NA	NA	300	NA
Vital status				
Dead	NA	173	457	57
Alive	NA	414	229	112

IDH, isocitrate dehydrogenase; MGMT, O^6^-methylguanine-DNA methyltransferase; codel, co-deletion.

NA represents No data.

### Expression of *IGHG1* Is Upregulated in Patients With Glioma

**Figure 1:**
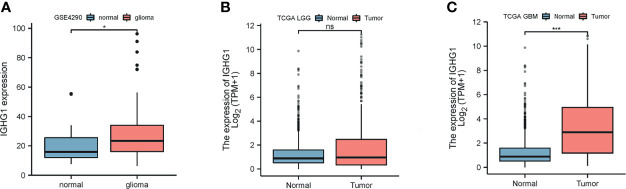
Differences in the expression of *IGHG1* between glioma and normal brain tissues. **(A)** In the GSE4290 dataset, the expression of *IGHG1* differed between glioma and normal brain tissues. **(B)** In The Cancer Genome Atlas (TCGA) cohort, there was no significant difference in the expression of *IGHG1* in lower grade glioma (LGG) and normal brain tissues. **(C)** The expression of *IGHG1* in glioblastoma (GBM) was significantly higher than that in normal brain tissue in TCGA. * represent P < 0.01. *** represent P < 0.0001 and ns, represent no significance.

We found that the expression of *IGHG1* in patients with glioma was higher than that in normal tissues from the GEO database (*p* < 0.01; **Figure 1A**). In TCGA, the expression of this gene showed a trend to be higher in low-grade glioma, but with no statistical significance (**Figure 1B**). However, *IGHG1* was upregulated remarkably in GBM (*p* < 0.001; **Figure 1C**). From the results, we drew the conclusion that the expression of *IGHG1* is upregulated in patients with glioma.

### Expression of *IGHG1* Is Correlated With Clinical Features That Predict Poor Prognosis

**Figure 2:**
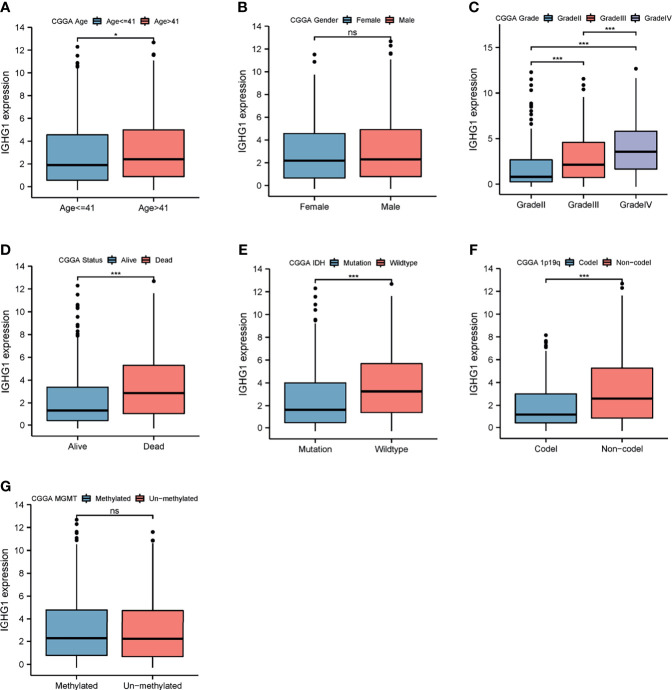
Differences in the expression of *IGHG1* among the different clinical characteristics in patients with glioma. Expression of *IGHG1* in different ages **(A)**, genders **(B)**, grades **(C)**, living state **(D)**, isocitrate dehydrogenase (IDH) status **(E)**, 1p19q status **(F)**, and *O*^6^-methylguanine-DNA methyltransferase (MGMT) status **(G)**. * represent P < 0.01. *** represent P < 0.0001 and ns, represent no significance.

To examine the expression of *IGHG1* in patients with different clinical characteristics, the CGGA database was used, which contains more details on the clinical features of the patients included in our study. The results showed that the expression of *IGHG1* was upregulated in patients over 41 years (*p* < 0.01; **Figure 2A**). There was no significant gender difference (**Figure 2B**). The expression level of *IGHG1* was upregulated coupled with grade promotion (*p* < 0.001; **Figure 2C**). Deceased patients had higher expression levels than did those who are alive (*p* < 0.001; **Figure 2D**). A high expression of *IGHG1* was found in patients with the molecular subtype IDH wild type and 1p19q non-codel (*p* < 0.001; **Figures 2E, F****)**, but there was no significance in the status of MGMT methylation (**Figure 2G**). In short, a high expression of *IGHG1* was correlated with the characteristics that predict poor prognosis.

### Glioma Patients With High Expression of *IGHG1* Had Poor Prognosis

**Figure 3:**
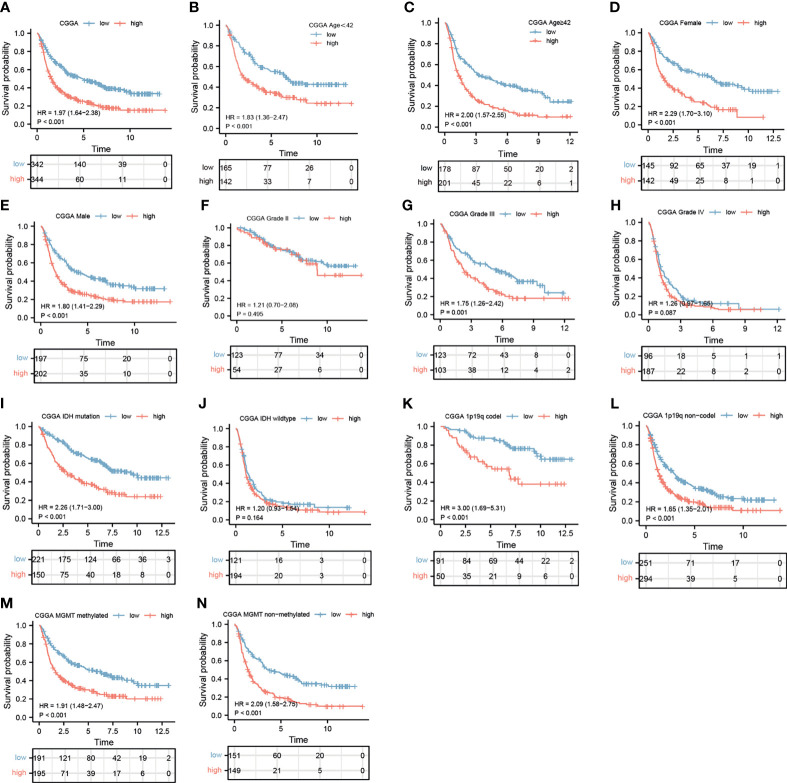
Prediction of the outcome of *IGHG1* in stratified patients in the Chinese Glioma Genome Atlas (CGGA) dataset. **(A)** Survival curve used to analyze overall survival (OS) in the low- and high-*IGHG1* groups in the CGGA dataset. **(A–N)** Survival analysis of the signature in patients stratified by age **(B, C)**, gender **(D, E)**, grade **(F, H)**, isocitrate dehydrogenase (IDH) status **(I, J)**, 1p19q status **(K, L)**, and *O*^6^-methylguanine-DNA methyltransferase (MGMT) promoter **(M, N)**.

Stratification analysis was programmed to evaluate the influence of *IGHG1* expression on the prognosis of glioma patients. The survival probability of patients with a high expression of *IGHG1* was poorer than that of those with a low expression, overall (HR = 1.97, 95%CI = 1.64–2.38, *p* < 0.001) (**Figure 3A**). We reached the same conclusions for patients younger than 41 years (HR = 1.83, 95%CI = 1.38–2.47, *p* < 0.001) (**Figure 3B**) and those over 41 years (HR = 2.01, 95%CI = 1.58–2.58, *p* < 0.001) (**Figure 3C**). The survival probability in patients with a high expression of the gene was worse in both females (HR = 2.29, 95%CI = 1.70–3.10, *p* < 0.001) (**Figure 3D**) and males (HR = 1.80, 95%CI = 1.41–2.29, *p* < 0.001) (**Figure 3E**). The survival probability of patients who were diagnosed with WHO grade III glioma (HR = 1.76, 95%CI = 1.26–2.42, *p* < 0.001) was significantly poor. But those with WHO grades II and IV did not reach the considered threshold (**Figures 3F–H**). Finally, patients with a high expression of *IGHG1* had a lower survival probability of reaching the threshold in the IDH mutation subgroup (HR = 2.26, 95%CI = 1.71–3.00, *p* < 0.001), the 1p19q codel subgroup (HR = 3.00, 95%CI = 1.69–5.31, *p* < 0.001), the 1p19q non-codel subgroup (HR = 1.65, 95%CI = 1.35–2.01, *p* < 0.001), the MGMT methylated subgroup (HR = 1.91, 95%CI = 1.40–2.47, *p* < 0.001), and the MGMT unmethylated subgroup (HR = 2.09, 95%CI = 1.08–2.70, *p* < 0.001), except for the IDH wild-type subgroup (**Figures 3I–N**). We also found that patients with a high expression of *IGHG1* had shorter PFI in TCGA cohort (LGG: HR = 1.44, 95%CI = 1.09–1.89, *p* = 0.009; GBM: HR = 1.41, 95%CI = 1.00–1.99, *p* = 0.053) (**Supplementary Figures S1A–D**). On the whole, patients with a high expression of *IGHG1* had poor prognosis.

### High *IGHG1* Expression Was Correlated With High Immune Infiltration

**Figure 4:**
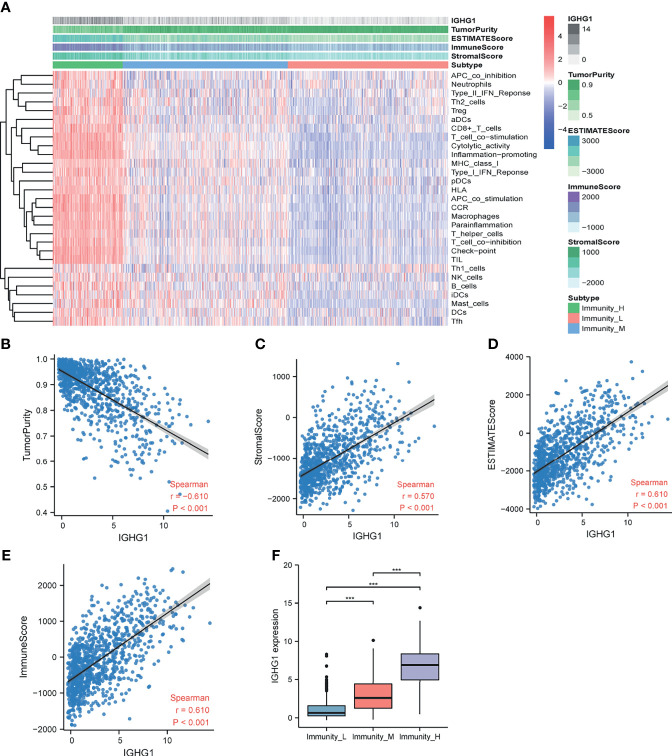
Immune infiltration patterns of the low- and high-*IGHG1* groups analyzed using single-sample gene set enrichment analysis (ssGSEA) methods in glioma from the Chinese Glioma Genome Atlas (CGGA) dataset. **(A)** Heatmap revealing the scores of immune cells in the low, medium, and high immunity groups. **(B–E)** Scatter plot showing the correlations between *IGHG1* and tumor purity, stromal score, ESTIMATE score, and immune score. **(F)** A high expression of *IGHG1* indicates more immune cell infiltration in glioma. *** represent P < 0.0001.

ssGSEA was performed to assess immune cell infiltration. Patients were clustered into low, moderate, and high immunity groups based on the state of immune cell infiltration. Analysis revealed that patients with more immunocyte infiltration also had a higher expression of *IGHG1*. The results are demonstrated in **Figure 4A**. There was also a distinct difference in the expressions of the genes among the groups. The expression level of *IGHG1* was negatively correlated with tumor purity (*r* = −0.610, *p* < 0.001) (**Figure 4B**), but it was positively correlated with the stromal score (*r* = 0.570, *p* < 0.001) (**Figure 4C**), ESTIMATE score (*r* = 0.610, *p* < 0.001) (**Figure 4D**), and immune score (*r* = 0.610, *p* < 0.001) (**Figure 4E**). In brief, *IGHG1* expression is closely relevant to the TME. A high expression of *IGHG1* indicates more immune cell infiltration in glioma (*p* < 0.001; **Figure 4F**).

### *IGHG1* Functions in Immune-Related Pathways in Glioma

**Figure 5:**
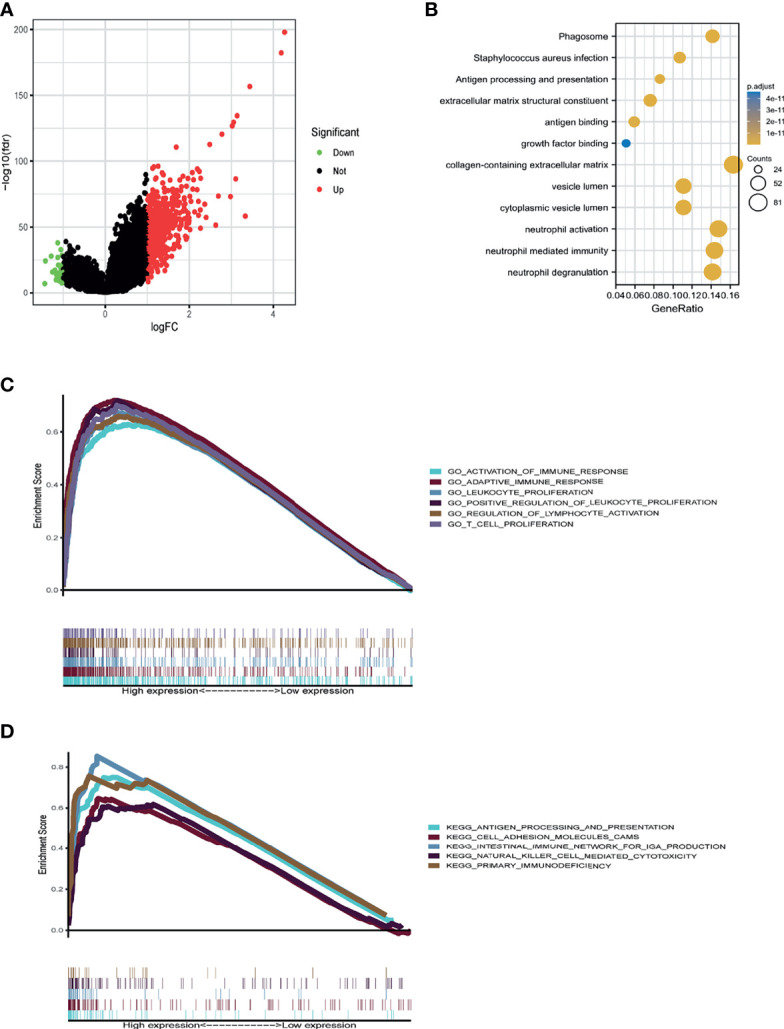
Database for Annotation, Visualization and Integrated Discovery (DAVID) and gene set enrichment analysis (GSEA) of the relevant mechanisms involved in *IGHG1*. **(A)** Volcano map showing the differentially expressed genes. **(B–D)** DAVID **(B)**, Kyoto Encyclopedia of Genes and Genomes (KEGG) **(C)**, and Gene Ontology (GO) **(D)** were used to analyze the relevant mechanisms.

To uncover the potential mechanisms of the functions of *IGHG1*, the CGGA database was analyzed to identify the DEGs. A volcano map was plotted for the DEGs (**Figure 5A**). DAVID analysis was carried out. A majority of the genes were related with immune-related functions, such as phagosome, antigen processing and presentation, extracellular matrix structural constituent, antigen binding, and collagen-containing extracellular matrix (**Figure 5B**). GO and KEGG analyses were performed using GSEA. The following were enriched in GO with thresholds of FDR < 0.05 and NES > 1: GO_ACTIVATION_OF_IMMUNE_RESPONSE, GO_ADAPTIVE_IMMUNE_RESPONSE, GO_LEUKOCYTE_PROLIFERATION, GO_REGULATION_OF_LYMPHOCYTE_ACTIVATION, and GO_T_CELL_PROLIFFERATION (**Figure 5C**). The following were enriched in KEGG analysis with thresholds of FDR < 0.05 and NES > 1: KEGG_ANTIGEN_PROCESSING_AND_PRESENTATION, KEGG_CELL_ADHESION_MELECULES_CAMS, KEGG_INTESTINAL_IMMUNE_NETWORK_FOR_IGA_PRODUCTION, and KEGG_PRIMARY_IMMUNODEFICIENCY (**Figure 5D**). To summarize, the same with ssGSEA, GO and KEGG analyses revealed that *IGHG1* plays a role in immune-related processes in glioma.

### Expression of Cancer-IgG Leads to Poor Prognosis by Immunohistochemistry Assay

**Figure 6:**
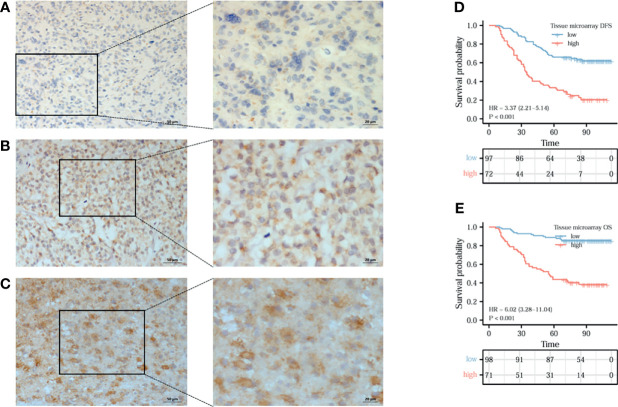
The expression of cancer-derived immunoglobulin G (cancer-IgG) in gliomas and its prognostic significance were analyzed by immunohistochemistry. **(A–C)** Cancer-IgG is weakly, moderately, and strongly positive in gliomas, respectively. **(D**, **E)** A high cancer-IgG expression in glioma was Analysis of the TMA showed that disease-free survival (DFS) (HR = 6.02, 95%CI =3.28–11.04, p < 0.001) **(D)** and OS (HR = 3.37, 95%CI = 2.21–5.14, p < 0.001) **(E)** were obviously poorer in patients with a high expression of cancer-IgG.

To back up our findings, immunohistochemistry assay was conducted with RP215, a mAb of cancer-IgG, using a TMA. **Figures 6A–C** show the weak staining, moderate staining, and strong staining intensities, respectively. Analysis of the TMA showed that OS (HR = 3.37, 95%CI = 2.21–5.14, *p* < 0.001) (**Figure 6D**) and disease-free survival (DFS) (HR = 6.02, 95%CI = 3.28–11.04, *p* < 0.001) (**Figure 6E**) were obviously poorer in patients with a high expression of cancer-IgG. In general, the expression of cancer-IgG represents poor prognosis.

## Discussion

Central nervous system cancer is a heterogeneous disease. Its new cases account for about 1.6% of the new tumors worldwide every year, and the mortality is about 2.5% (17). Gliomas accounting for 80% of central nervous system tumors have the characteristics of heterogeneity and complexity. Patients with glioma, especially GBM, have poor prognosis with a median survival of <2 years (5). From the initial morphological classification to the latest molecular classification in 2021, the accuracy of glioma diagnosis and prediction has been greatly improved. However, only a few of the available molecular markers truly influence clinical decision-making and treatments such as MGMT promoter methylation, co-deletion of 1p and 19q, and *IDH1*/*IDH2* mutations (18). Therefore, it is urgent to understand in depth the pathogenesis of glioma, discover new molecular targets, and develop new treatment methods.

When it comes to IgG, which is composed of two heavy chains and two light chains, we firstly hold that it is an antibody secreted by B cells that has a protective effect on the body. However, there is a growing view that tumor cells can also produce IgG by themselves, as cancer-IgG. Cancer-IgG has been widely studied in many specified epithelial tumors, such as breast cancer (6), prostate cancer (19), and bladder cancer (20). More and more evidence also showed that cancer-IgG promotes the occurrence and development and the immune escape of tumors. But the role of cancer-IgG in tumorigenesis is complex and, in glioma, is poorly understood.

In our study, we firstly analyzed the expression of *IGHG1*, the gene encoding the heavy chain of IgG, in glioma with bioinformatics methods. The expression level of *IGHG1* in patients with glioma was apparently upregulated from the GEO and TCGA databases, especially in GBM. The OS of patients with a higher expression of *IGHG1* had worse prognosis compared with those with a lower expression. Similar consequences have been found in some epithelial cancers. Xinyu et al. provided novel evidence that *IGHG1* acted as an oncogene by promoting gastric cancer cellular proliferation, migration, and chemoresistance (21). Jing et al. demonstrated that *IGHG1* was increased in prostate cancer tissues and promoted cell growth through activating the MEK/ERK/c-Myc pathway (22). In order to further verify the role of *IGHG1* in glioma, we performed stratified analysis. The results showed that patients with the molecular subtype IDH wild type and 1p19q non-codel had a higher expression of *IGHG1*. As is known, IDH wild type and 1p19q non-codel represent poor prognosis (23). Furthermore, our analysis showed that patients who were diagnosed with WHO grade IV glioma had the highest expressions of *IGHG1*. WHO grade II glioma patients had the lowest expressions. The expression of *IGHG1* was positively relevant to tumor grade and could predict adverse prognosis. It turns out that a high expression of *IGHG1* was associated with some of the molecular subtypes mentioned previously that represent bad prognosis.

RP215 is a mAb that specifically recognizes the sialylation site of the heavy chain of cancer-IgG (24). In the present study, we used RP215 for immunohistochemical staining, which distinguishes IgG produced by B cells. Similar to the results of the bioinformatics analysis, it was shown that a high level of cancer-IgG is significantly related to poor prognosis in glioma. Patients with a higher expression of cancer-IgG have shorter DFS. In addition, cancer-IgG expression was shown to be a powerful prognostic marker for survival. Previous researchers have discovered that cancer-IgG is an independent poor prognostic factor, as also suggested by our findings in glioma studies. Ming et al. found that a high cancer-IgG expression in pancreatic ductal adenocarcinoma and parathyroid carcinoma was related to poor DFS and OS (25, 26). Jiang et al. studied cancer-IgG in colorectal cancer. They found that the overexpression of cancer-IgG in colorectal cancer patients led to poor prognosis (27).

Many studies have concentrated on the mechanisms of cancer-IgG in carcinogenesis. Qiu et al. firstly discovered that cancer-IgG has growth factor-like activity (28). Later studies also proved this view and further found that cancer-IgG can also play the role of an oncogene through the AKT, FAK, SOX2, and other signaling pathways in cancer cells (8, 9, 19, 29). Interacting cells in the TME are considered to regulate the characteristics of cancers, such as uncontrolled proliferation, malignant metastasis, and chemoradiotherapy resistance (30). Recent studies have shown that cancer cells could secrete cancer-IgG into the TME that binds to sialic acid-binding immunoglobulin-type lectins (Siglecs) on effector CD4^+^ and CD8^+^ T cells. Then, cancer-IgG is secreted into the TME and promotes tumor cell immune escape (11). Xiaoyan et al. discovered that *IGHG1* in pancreatic carcinomas is associated with immune evasion (31). In our study, we explored the role of *IGHG1* in the glioma microenvironment with ssGSEA. The results illustrated that patients with a high expression of *IGHG1* were clustered into a high immunity group and those with a low expression into a low immunity group. A high expression of *IGHG1* was correlated with more immunocyte infiltration. Immune cells including the microglia and peripheral macrophages, granulocytes, myeloid-derived suppressor cells, and T lymphocytes infiltrate into the glioma. In the microenvironment of glioma, the infiltration of microglia/macrophages and myeloid-derived suppressor cells was negatively correlated with prognosis (32). Combined with our findings, this suggests that *IGHG1* could play a role in immune-related processes, leading to poor OS. We made use of GSEA and DAVID to verify the results of the ssGSEA. We confirmed that *IGHG1* played a role in immune-related pathways. Defects of antigen processing pathways are relevant to malignant transformation, leading to the loss of major histocompatibility complex (MHC I) in cancer cells, which is one of the mechanisms of immune escape (33). Our results suggested that the expressions of *IGHG1* and cancer-IgG could induce immune escape, contributing to poor survival.

In this study, we found that the expressions of *IGHG1*/cancer-IgG were higher in glioma with poor prognosis. In addition, *IGHG1*/cancer-IgG were closely related to immune cell infiltration in the glioma microenvironment. Together, *IGHG1*/cancer-IgG are promising biomarkers of diagnosis and treatment in patients with glioma. However, the conclusion of this article, only from bioinformatics analysis and immunohistochemistry, needs to be further verified in *in vivo* and *in vitro* experiments. The detailed mechanism needs to be explored in further studies.

## Data Availability Statement

The datasets presented in this study can be found in online repositories. The names of the repository/repositories and accession number(s) can be found in the article/**Supplementary Material**.

## Ethics Statement

Ethical review and approval was not required for the study on human participants, in accordance with the local legislation and institutional requirements. Written informed consent for participation was not required for this study, in accordance with the national legislation and the institutional requirements.

## Author Contributions

XX was responsible for the overall design of this study. GW analyzed the data and edited the manuscript. HL and JP were mainly responsible for data analysis. TY contributed to the study guidance of R software. HZ was responsible for immunohistochemical staining and scoring. XH and LS provided R language modification. LB revised the discussion of the article. All authors contributed to the article and approved the submitted version.

## Funding

This study was supported by the Key R&D Program of Hebei Province (19277737D).

## Conflict of Interest

The authors declare that the research was conducted in the absence of any commercial or financial relationships that could be construed as a potential conflict of interest.

## Publisher’s Note

All claims expressed in this article are solely those of the authors and do not necessarily represent those of their affiliated organizations, or those of the publisher, the editors and the reviewers. Any product that may be evaluated in this article, or claim that may be made by its manufacturer, is not guaranteed or endorsed by the publisher.

## Supplementary Material

Supplementary Figure 1 **(A, B)** Survival analysis of low- and high-IGHG1 patients in the TCGA cohorts.
