# Supplementary figures and images for: Upregulated Expression of Cancer-Derived Immunoglobulin G Is Associated With Progression in Glioma
Source: Front Oncol. 2021 Oct 25;11:758856. doi: 10.3389/fonc.2021.758856 (PMC8574069; doi:10.3389/fonc.2021.758856)

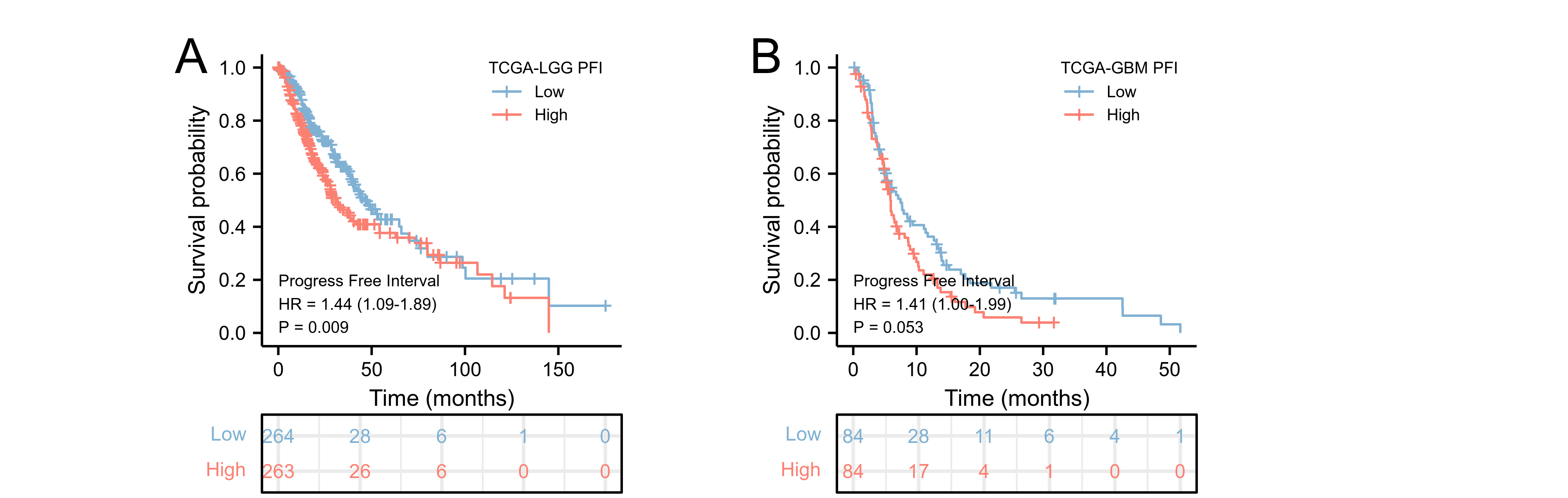

Supplement: Supplementary Figure 1 — (A, B) Survival analysis of low- and high-IGHG1 patients in the TCGA cohorts. [file Image_1.tiff]
